# Case report: Whole exome sequencing identified a novel mutation (p.Y301H) of *MAF* in a Chinese family with congenital cataracts

**DOI:** 10.3389/fmed.2024.1332992

**Published:** 2024-02-29

**Authors:** Zhao-Jing Lin, Jie-Yi Long, Juan Li, Fang-Na Wang, Wei Chu, Lei Zhu, Ya-Li Li, Liang-Liang Fan

**Affiliations:** ^1^Department of Anesthesiology, The Second Xiangya Hospital of Central South University, Changsha, China; ^2^Department of Cell Biology, School of Life Sciences, Central South University, Changsha, China; ^3^Department of Reproductive Genetics, Hebei General Hospital, Shijiazhuang, China; ^4^Department of Obstetrics and Gynecology, Ordos Central Hospital, Ordos, China

**Keywords:** congenital cataract, inherited cataract, *MAF*, whole exome sequencing, missense mutation

## Abstract

**Background:**

Congenital cataracts stand as the primary cause of childhood blindness globally, characterized by clouding of the eye’s lens at birth or shortly thereafter. Previous investigations have unveiled that a variant in the *V-MAF avian musculoaponeurotic-fibrosarcoma oncogene homolog* (MAF) gene can result in Ayme-Gripp syndrome and solitary cataract. Notably, MAF mutations have been infrequently reported in recent years.

**Methods:**

In this investigation, we recruited a Chinese family with non-syndromic cataracts. Whole exome sequencing and Sanger sequencing were applied to scrutinize the genetic anomaly within the family.

**Results:**

Through whole exome sequencing and subsequent data filtration, a new mutation (NM_005360, c.901T>C/p.Y301H) in the *MAF* gene was detected. Sanger sequencing validated the presence of this mutation in another affected individual. The p.Y301H mutation, situated in an evolutionarily preserved locus, was not detected in our 200 local control cohorts and various public databases. Additionally, multiple bioinformatic programs predicted that the mutation was deleterious and disrupted the bindings between *MAF* and its targets.

**Conclusion:**

Hence, we have documented a new *MAF* mutation within a Chinese family exhibiting isolated congenital cataracts. Our study has the potential to broaden the spectrum of *MAF* mutations, offering insights into the mechanisms underlying cataract formation and facilitating genetic counseling and early diagnosis for congenital cataract patients.

## Introduction

Congenital cataracts, an ophthalmic disease presented at birth or developing in infancy, can lead to a clouding in the lens of the eye that can cause blurry vision or blindness (1–3). As the predominant cause of visual impairment and blindness among children, congenital cataracts affect approximately 200,000 children worldwide, with an estimated prevalence ranging from three to six per 10,000 live births (4, 5). The current studies believe that multiple factors including genetics, infections, radiation exposure, toxic agents, and metabolic disturbance underlie the occurrence of congenital cataracts (3). To date, over 40 genes responsible for the molecular etiology of isolated or syndromic congenital cataracts, featuring autosomal dominant or autosomal recessive inheritance patterns, have been identified (1, 6).

The *V-MAF avian musculoaponeurotic-fibrosarcoma oncogene homolog* (*MAF*) gene, also known as MAF bZIP transcription factor (OMIM#177075), is situated on chromosome 16q23.2, spanning approximately 6.9 kilobases (7). As a DNA-binding transcription factor with a leucine zipper motif that can control the activity of target genes, MAF can serve as a transcriptional activator or repressor, regulating diverse cellular processes, including embryonic lens fiber cell development, heightened T-cell susceptibility to apoptosis, and chondrocyte terminal differentiation (8–10). In 2002, Jamieson et al. (11) initially reported the association of *MAF* with cataracts, ocular anterior segment dysgenesis, and coloboma. Subsequently, approximately 30 *MAF* mutations have been detected in patients with Ayme-Gripp syndrome and isolated cataracts (7).

Here, we recruited a Chinese family affected by isolated congenital cataracts. Utilizing whole exome sequencing and Sanger sequencing, we sought to uncover the genetic abnormalities in the affected individuals.

## Case presentation

**Figure 1:**
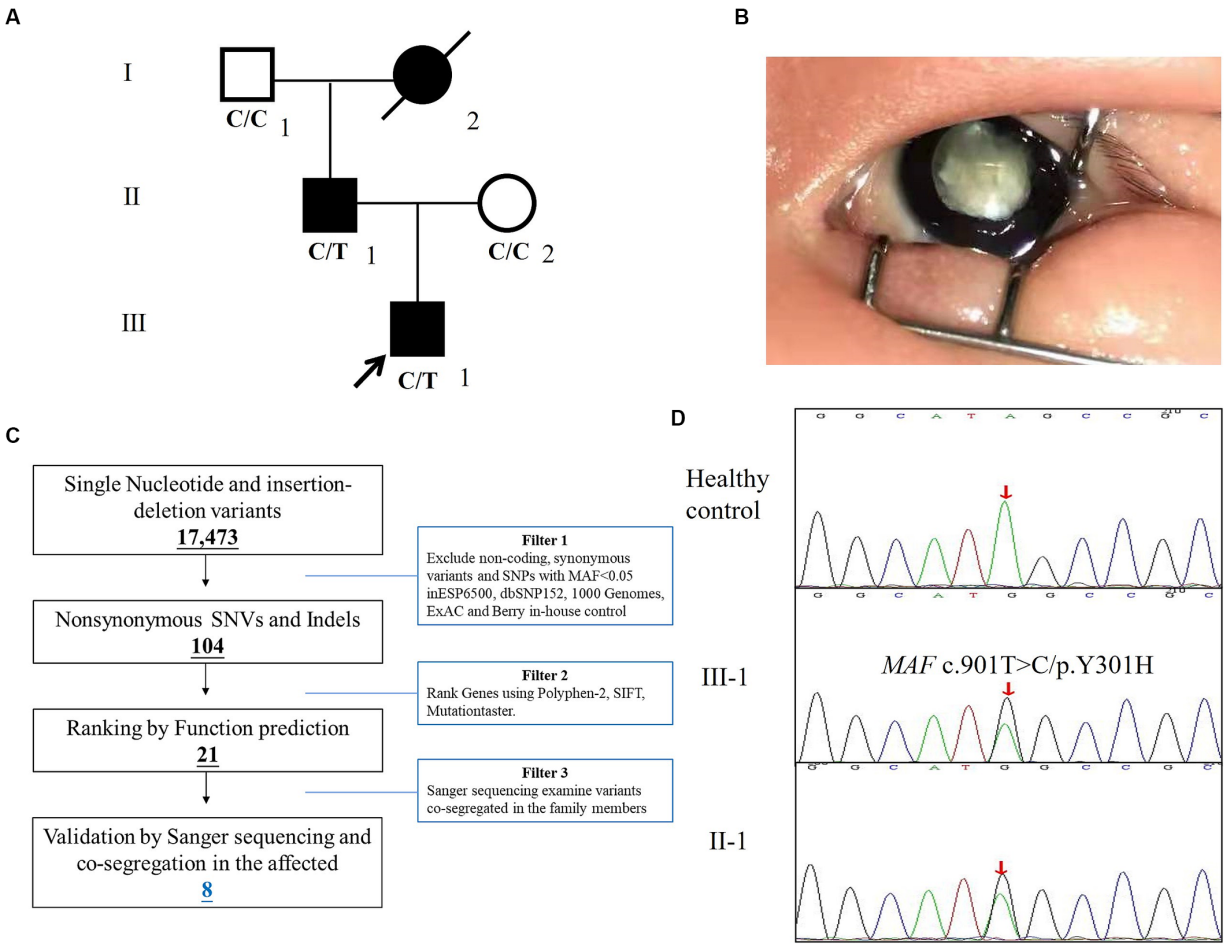
The clinical and genetic analysis of the family with congenital cataracts. **(A)** Pedigree of the family. White circles/squares are unaffected family members, and the arrow indicates the proband. **(B)** The right eye of the proband. **(C)** Schematic representation of the filter strategies employed in our study. **(D)** Sanger DNA sequencing chromatogram demonstrates the heterozygosity for an *MAF* missense mutation (c.901T>C/p.Y301H) in the family.

Here, we enrolled the family from Hebei province, China (Figure 1A). The affected proband, a four-year-old boy was admitted to Hebei General Hospital and diagnosed as a total congenital cataract of the right eye (Figure 1B). A medical history survey suggested that the patient suffered from poor vision in dim light at approximately 3 years old but did not arouse parents’ attention, and the symptoms of the child developed to lens complete opacification from pupil gray appearance quickly during this year. Finally, the patient agreed to undergo surgery for cataract extraction and implantation for the posterior chamber intraocular lens, and the eye vision is gradually recovering. A study of the family history indicated that his father also suffered from cataracts and underwent cataract extraction in infancy. The proband’s grandmother was blind and died several years ago.

### Laboratory investigations

Whole peripheral blood samples of the patient and his family were obtained and stored in EDTA/citrate tubes. Genomic DNA was extracted by GenElute blood genomic DNA extraction kit (Sigma-Aldrich, NA 2010). Whole Exome sequencing (WES) was mainly conducted by Berry Genomics (Beijing, China) and Agilent SureSelect Human All Exon V6 kits and Illumina HiSeq2500 (Illumina Inc., San Diego, United States) and matched to Human Reference Genome (hg19). GATK online software^1^ was used to detect SNP, Indel, and variants, and the Annovar (Annotate Variation) tool was used to functionally annotate genetic variant results in detail.

The strategies of data filtering are as follows (12, 13): (a) non-coding and synonymous variants, SNPs, or frameshift-causing INDELs with an alternative allele frequency >0.05 in the NHLBI Exome Sequencing Project Exome Variant Server (ESP6500), dbSNP152,^2^ the 1,000 Genomes project,^3^ the ExAC database,^4^ or in-house exome databases of BerryGenomics (2000 exomes) were excluded; (b) the filtered SNVs and INDELs, predicted by SIFT,^5^ Polyphen2,^6^ and MutationTaster^7^ to be causing damage remained; (c) co-segregation analysis was conducted in each family.

The filtered mutations validation and co-segregation analysis were performed by Sanger sequencing. The primer pairs (the sequence of primers will be provided upon request) were designed by Primer 5. The sequences of the primers were: 5′-3′ TCAGCAAGGAGGAGGTGAT and 3′-5′ CTGCTCACCAACTTCTCGTATT. The sequence of the polymerase chain reaction products was determined using the ABI 3100 Genetic Analyzer (ABI, United States).

**Figure 2:**
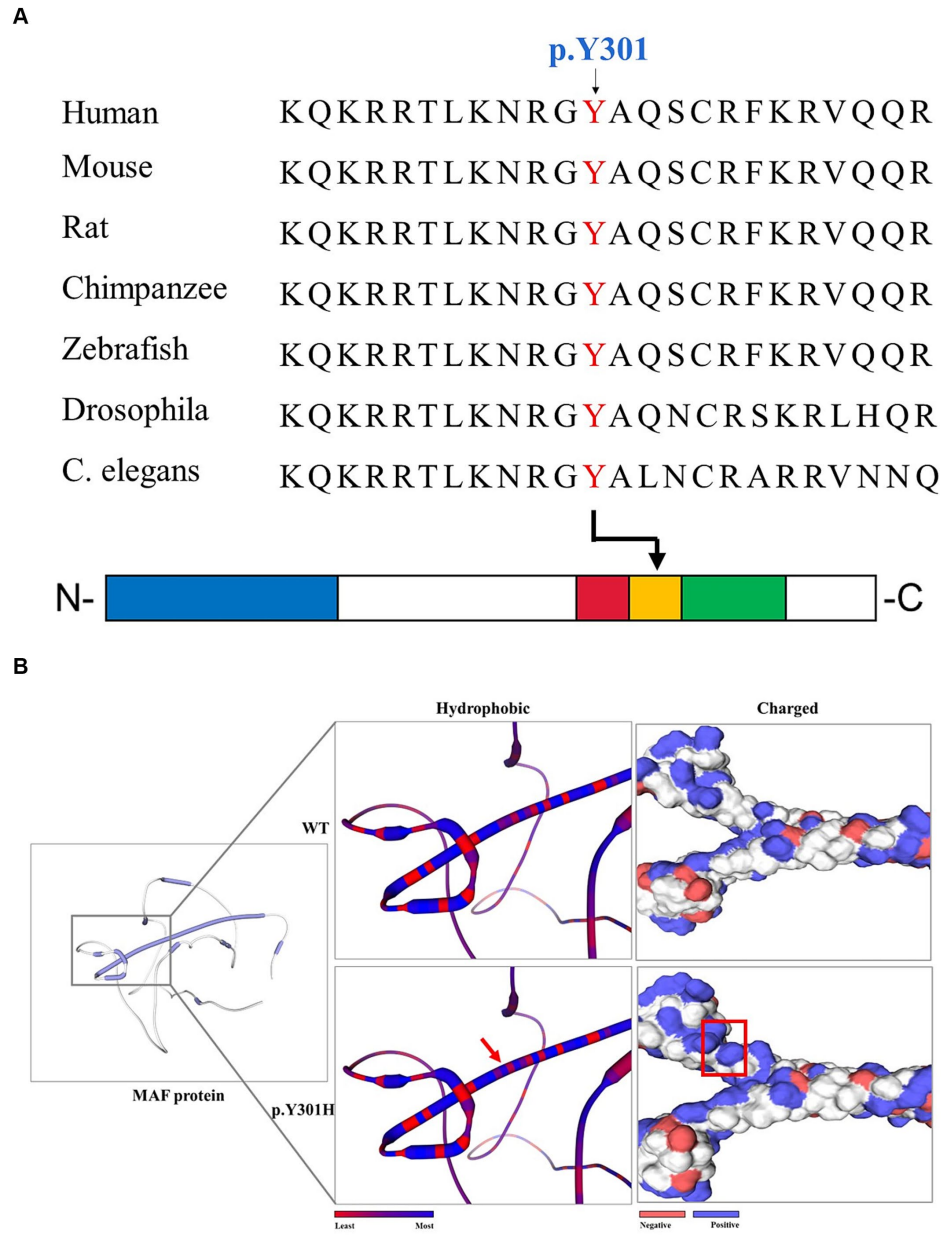
The bioinformatics analysis of the *MAF* p.Y301H mutation. **(A)** Alignment of multiple MAF protein sequences across species. The Y301-affected amino acid is located in the highly conserved amino acid region in different mammals (from Ensembl). The red column shows the Y301 site. Blue square: transactivation domain. Red square: extended homology region. Orange square: basic region motif. Green square: leucine zipper motif. **(B)** The hydrophobic surface area and surface charge of the WT and mutated MAF were predicted by SWISS-MODEL and alphafold2. The red arrow and red square indicate the differences between WT and mutated protein.

After the abovementioned data filtering and Sanger sequencing validation (Figure 1C), a new mutation (NM_005360, c.901T>C/p.Y301H) of *MAF* was discerned in the proband (Table 1). No other meaningful mutations related to cataracts were detected. Co-segregation analysis indicated that the new mutation was present in the affected family members and was not detected in unaffected family members and healthy controls as well as 200 local control cohorts (Figure 1D). Three programs for analyzing protein functions, polyphen2, SIFT, and MutationTaster, predicted that the p.Y301H variants are probably damaging (1.0), deleterious (0.00), and disease-causing (0.99), respectively. Cross-species alignment analysis of MAF amino acid sequences revealed that this mutated site was highly evolutionarily conserved (Figure 2A). Swiss-Model and alphafold2 online software (14) found that the p.Y301H mutation changed the surface hydrophobic area and surface charge of MAF (Figure 2B).

**Table 1:** The gene list of Sanger sequencing validation and co-segregation analysis.

Gene	Chromosome position	Variant	Genotype	ACMG analysis	Diseases in OMIM
RRM2B	Chr 8:103220407	NM_015713: c.1010T>C, p.M337T	Het	Uncertain significance	AR, mitochondrial DNA depletion syndrome; AR, rod-cone dystrophy and sensorineural deafness
DNA2	Chr 10:70209793	NM_001080449: c.931C>T, p.R311C	Het	Uncertain significance	AR, Seckel syndrome; AD, progressive external ophthalmoplegia
AGPS	Chr 2:178257534	NM_003659: c.17C>G, p.A6G	Het	Uncertain significance	AR, rhizomelic chondrodysplasia punctata
GDF3	Chr 12:7842818	NM_020634: c.751G>A, p.A251T	Het	Uncertain significance	AD, microphthalmia
RDH11	Chr 14:68162398	NM_016026: c.23T>C, p.L8P	Het	Uncertain significance	AR, retinal dystrophy, juvenile cataracts, and short stature syndrome
MAF	Chr 16:79632899	NM_005360: c.901T>C, p.Y301H	Het	Likely pathogenic	AD, cataract; AD, Ayme-Gripp syndrome
COL1A2	Chr 6:33154514	NM_080680: c.688G>T, p.G230W	Het	Uncertain significance	AD, Ehlers-Danlos syndrome; AD, osteogenesis imperfecta
CRB1	Chr 1:197398616	NM_201253: c.2714G>A, p.R905Q	Het	Uncertain significance	AR, leber congenital amaurosis; AD, pigmented paravenous chorioretinal atrophy; AR, Retinitis pigmentosa

AD, autosomal dominant; AR, autosomal recessive; Het, heterozygotes.

## Discussion

Congenital cataracts represent a highly diverse ocular disorder both clinically and genetically (3). Mutations in MAF have been linked to various types and severities of human congenital cataracts (Table 2) (32). MAF proteins play a pivotal role in eyes and lens development, regulating the expression of crystallin genes, MIP (major intrinsic protein of the ocular lens fiber membrane), and other genes expressed in lens fiber cells from the formation of the lens pre-placode to the development of lens fiber cells and lens epithelium (8–10). In C57BL/6J mice, homozygous deletion of *MAF* results in embryonic lethality, but the lens fiber cell MAF condition knockout mice (MAF^ΔTAM^) develops abnormal lens structure, and the expression of crystallin genes in MAF^ΔTAM^ mice eyes are reduced compared to WT mice (33). The mice data were consistent with human genetic studies, highlighting the essential role of MAF in the differentiation and cell cycle arrest of lens fiber cells. Although the first mutation of MAF was reported in 2002 (11), the MAF novel mutation was rarely reported in recent years. Here, we identified a new variant (NM_005360, c.901T>C/p.Y301H) of MAF in a Chinese family with congenital cataracts via whole exome sequencing and Sanger sequencing. Our study expanded the mutation spectrum of *MAF* and further proved that mutations in *MAF* may lead to congenital cataracts.

**Table 2:** The summary of reported mutations in *MAF* gene.

No	Mutation	Domain	Phenotypes	Reference
1	p.Ser54Leu	TAD	Cataracts	Niceta et al. (15)
2	p.Ser54Trp	TAD	Ayme-Gripp syndrome	Amudhavalli et al. (16)
3	p.Ser57Phe	TAD	Ayme-Gripp syndrome	Niceta et al. (17)
4	p.Thr58Ala/Ile	TAD	Cataracts	Niceta et al. (15)
5	p.Pro59Leu/His	TAD	Cataracts	Niceta et al. (15)
6	p.Pro59Arg	TAD	Ayme-Gripp syndrome	Javadiyan et al. (18)
7	p.Thr62Arg	TAD	Cataracts	Niceta et al. (15)
8	p.Pro63Arg	TAD	Ayme-Gripp syndrome	Amudhavalli et al. (16)
9	p.Ser66Trp	TAD	Ayme-Gripp syndrome	Amudhavalli et al. (16)
10	p.Ser66Leu	TAD	Ayme-Gripp syndrome	Niceta et al. (17)
11	p.Pro69Arg	TAD	Cataracts	Niceta et al. (15)
12	p.Ala169Ser	EHR	Cataracts	Liu et al. (19)
13	p.Phe261Ser	EHR	Cataracts	Jackson et al. (20)
14	p.Ser270Tyr	EHR	Cataracts	Dudakova et al. (21)
15	p.Val271Glu	EHR	Cataracts	Si et al. (7)
16	p.Glu273Asp	EHR	Cataracts	Ma et al. (22)
17	p.Arg288Pro	BR	Cataracts	Jamieson et al. (11)
18	p.Arg294Trp	BR	Cataracts	Sun et al. (23)
19	p.Lys297Arg	BR	Cataracts	Vanita et al. (24)
20	p.Asn298Tyr	BR	Cataracts	Patel et al. (25)
21	p.Arg299Ser	BR	Cataracts	Hansen et al. (26)
22	p.Tyr301His	BR	Cataracts	This study
23	p.Gln303Pro	BR	Cataracts	Narumi et al. (27)
24	p.Cys305Trp	BR	Cataracts	Ma et al. (22)
25	p.Arg306Gly	BR	Cataracts	Ma et al. (28)
26	p.Glu317Gly	LZM	Cataracts	Li et al. (29)
27	p.Lys320Glu	LZM	Cataracts	Hansen et al. (30)
28	p.Pro366Argfs*8	LZM	Cataracts	Ziats et al. (31)

TAD, transactivation domain; HER, extended homology region; BR, basic region motif; LZM, leucine zipper motif.

The MAF protein consists of an N-terminal transactivation domain with a regulatory function and a C-terminal DNA binding domain (18). The C-terminal domain is further divided into three conserved regions (Figure 2A): extended homology region, basic region motif, and leucine zipper motif (15, 34). Earlier studies have indicated that the N-terminal variants may result in Ayme-Gripp syndrome which presents with cataracts, hearing loss, epilepsy, intellectual disability, etc., while most of the C-terminal mutation carriers only showed ocular diseases such as cataracts (Table 2) (7). Here, the mutation (p.Y301H) is also situated in the C-terminal of MAF, which provides more evidence that C-terminal mutations are responsible for isolated cataracts.

Furthermore, the p.Y301H mutation is in the BR motif of the C-terminal, which is the mutational hot spot region of MAF (7). Previous studies have suggested that the BR motif is responsible for binding the specific target promoters of crystallin genes including CRYGA, CRYAA, CRYBA1, and CRYBA4, which are associated with inherited cataracts (32, 35). Additionally, ChIP-seq studies have identified several non-crystallin genes crucial for maintaining lens transparency as direct targets of MAF (36, 37). Hence, the p.Y301H mutation may disrupt the bindings between MAF and the target genes such as crystallin genes, and finally disrupt the expression of promoters of crystallin genes, leading to congenital cataracts. Following the ACMG guideline (38), the p.Y301H mutation is likely pathogenic (PM1 + PM2 + PP1 + PP2 + PP3).

Congenital cataracts persist as a leading cause of global blindness, and early surgical intervention, prolonged postoperative amblyopia training, and visual reconstruction constitute the primary therapeutic approaches for the disease (39, 40). Early diagnosis is paramount, particularly in the era of precision medicine, where technologies such as PCR, Sanger sequencing, high-throughput sequencing, and gene editing have facilitated the discovery of numerous pathogenic genes linked to congenital cataract development. This advancement enhances our understanding of the disease’s pathogenesis and lays the groundwork for genetics-based treatments (6, 28). In the future, we can develop a genetic detection panel that contains all the reported mutations of *MAF* including the p.Y301H, and the panel may contribute to the genetic counseling and early diagnosis of congenital cataract patients.

## Conclusion

Hence, we detected a new mutation (NM_005360, c.901T>C/p.Y301H) of *MAF* in a Chinese family with congenital cataracts by employing whole exome sequencing and Sanger sequencing. Our study not only explores the genetic lesion of the family and broadens the spectrum of *MAF* mutations but also confirms that the *MAF* mutation was linked to non-syndromic total congenital cataracts and facilitates genetic counseling and early diagnosis for congenital cataract patients. Certainly, there were still some limitations in this study, for example, the sample size was small and population specificity was not excluded.

## Data availability statement

The original contributions presented in the study are included in the article/supplementary material, further inquiries can be directed to the corresponding authors.

## Ethics statement

The studies involving humans were approved by the Ethics Committee of Hebei General Hospital. The studies were conducted in accordance with the local legislation and institutional requirements. Written informed consent for participation in this study was provided by the participants’ legal guardians/next of kin. Written informed consent was obtained from the individual(s), and minor(s)’ legal guardian/next of kin, for the publication of any potentially identifiable images or data included in this article. Written informed consent was obtained from the participant/patient(s) for the publication of this case report.

## Author contributions

Z-JL: Data curation, Formal analysis, Writing – original draft. J-YL: Formal analysis, Writing – review & editing. JL: Resources, Writing – review & editing. F-NW: Resources, Writing – review & editing. WC: Resources, Writing – review & editing. LZ: Formal analysis, Funding acquisition, Writing – original draft. Y-LL: Funding acquisition, Resources, Writing – original draft. L-LF: Funding acquisition, Supervision, Writing – original draft, Writing – review & editing.

## Footnotes

^1^www.broadinstitute.org/gatk

^2^http://www.ncbi.nlm.nih.gov/projects/SNP/index.html

^3^http://www.1000genomes.org/

^4^http://exac.broadinstitute.org

^5^http://sift.jcvi.org/

^6^http://genetics.bwh.harvard.edu/pph2/

^7^http://www.mutationtaster.org/

## References

[1] Berry V Georgiou M Fujinami K Quinlan R Moore A Michaelides M. Inherited cataracts: molecular genetics, clinical features, disease mechanisms and novel therapeutic approaches. Br J Ophthalmol. (2020) 104:1331–7. doi: 10.1136/bjophthalmol-2019-315282, PMID: 32217542

[2] Gali HE Sella R Afshari NA. Cataract grading systems: a review of past and present. Curr Opin Ophthalmol. (2019) 30:13–8. doi: 10.1097/ICU.0000000000000542 30489359

[3] Liu YC Wilkins M Kim T Malyugin B Mehta JS. Cataracts. Lancet. (2017) 390:600–12. doi: 10.1016/S0140-6736(17)30544-5 28242111

[4] Hashemi H Pakzad R Yekta A Aghamirsalim M Pakbin M Ramin S. Global and regional prevalence of age-related cataract: a comprehensive systematic review and meta-analysis. Eye. (2020) 34:1357–70. doi: 10.1038/s41433-020-0806-3, PMID: 32055021 PMC7376226

[5] Medsinge A Nischal KK. Pediatric cataract: challenges and future directions. Clin Ophthalmol. (2015) 9:77–90. doi: 10.2147/OPTH.S59009, PMID: 25609909 PMC4293928

[6] Li J Chen X Yan Y Yao K. Molecular genetics of congenital cataracts. Exp Eye Res. (2020) 191:107872. doi: 10.1016/j.exer.2019.107872 31770519

[7] Si N Song Z Meng X Li X Xiao W Zhang X. A novel MAF missense mutation leads to congenital nuclear cataract by impacting the transactivation of crystallin and noncrystallin genes. Gene. (2019) 692:113–8. doi: 10.1016/j.gene.2019.01.011, PMID: 30659945

[8] Cvekl A Mcgreal R Liu W. Lens development and crystallin gene expression. Prog Mol Biol Transl Sci. (2015) 134:129–67. doi: 10.1016/bs.pmbts.2015.05.001 26310154

[9] Ring BZ Cordes SP Overbeek PA Barsh GS. Regulation of mouse lens fiber cell development and differentiation by the *Maf* gene. Development. (2000) 127:307–17. doi: 10.1242/dev.127.2.307 10603348

[10] Xie Q Mcgreal R Harris R Gao CY Liu W Reneker LW. Regulation of c-Maf and alphaA-Crystallin in ocular lens by fibroblast growth factor Signaling. J Biol Chem. (2016) 291:3947–58. doi: 10.1074/jbc.M115.705103, PMID: 26719333 PMC4759173

[11] Jamieson RV Perveen R Kerr B Carette M Yardley J Heon E. Domain disruption and mutation of the bZIP transcription factor, MAF, associated with cataract, ocular anterior segment dysgenesis and coloboma. Hum Mol Genet. (2002) 11:33–42. doi: 10.1093/hmg/11.1.33, PMID: 11772997

[12] Fang GM Miao JX Peng Y Zhai YF Wang CC Zhao XY. Identification of three *FBN1* mutations in Chinese patients with typical or incomplete Marfan syndrome by whole-exome sequencing. Cardiovasc Innov Appl. (2020) 5:19–26. doi: 10.15212/CVIA.2019.0576

[13] Yu R Liu L Li YL Fan LL. MITF p.Arg217Thr variant identified in a Han Chinese family with Tietz/Waardenburg syndrome. Biomed Res Int. (2021) 2021:4381272. doi: 10.1155/2021/4381272 33506017 PMC7815406

[14] Lu G Zhang Y Xia H He X Xu P Wu L. Identification of a *de novo* mutation of the FOXG1 gene and comprehensive analysis for molecular factors in Chinese FOXG1-related encephalopathies. Front Mol Neurosci. (2022) 15:1039990. doi: 10.3389/fnmol.2022.1039990, PMID: 36568277 PMC9768341

[15] Niceta M Stellacci E Gripp KW Zampino G Kousi M Anselmi M. Mutations impairing GSK3-mediated MAF phosphorylation cause cataract, deafness, intellectual disability, seizures, and a down syndrome-like Facies. Am J Hum Genet. (2015) 96:816–25. doi: 10.1016/j.ajhg.2015.03.001, PMID: 25865493 PMC4570552

[16] Amudhavalli SM Hanson R Angle B Bontempo K Gripp KW. Further delineation of Aymé-Gripp syndrome and use of automated facial analysis tool. Am J Med Genet A. (2018) 176:1648–56. doi: 10.1002/ajmg.a.38832 30160832

[17] Niceta M Barbuti D Gupta N Ruggiero C Tizzano EF Graul-Neumann L. Skeletal abnormalities are common features in Aymé-Gripp syndrome. Clin Genet. (2020) 97:362–9. doi: 10.1111/cge.13651 31600839

[18] Javadiyan S Craig JE Sharma S Lower KM Casey T Haan E. Novel missense mutation in the bZIP transcription factor, MAF, associated with congenital cataract, developmental delay, seizures and hearing loss (Ayme-Gripp syndrome). BMC Med Genet. (2017) 18:52. doi: 10.1186/s12881-017-0414-7, PMID: 28482824 PMC5422868

[19] Liu Y Liu X Qin D Zhao Y Cao X Deng X. Clinical Utility of Next-Generation Sequencing for Developmental Disorders in the Rehabilitation Department: Experiences from a Single Chinese Center. J Mol Neurosci. (2021) 71:845–53. doi: 10.1007/s12031-020-01707-4 32959227

[20] Jackson D Malka S Harding P Palma J Dunbar H Moosajee M. Molecular diagnostic challenges for non-retinal developmental eye disorders in the United Kingdom. Am J Med Genet C Semin Med Genet. (2020) 184:578–89. doi: 10.1002/ajmg.c.31837 32830442 PMC8432170

[21] Dudakova L Stranecky V Ulmanova O Hlavova E Trková M Vincent AL. Segregation of a novel p.(Ser270Tyr) MAF mutation and p.(Tyr56∗) CRYGD variant in a family with dominantly inherited congenital cataracts. Mol Biol Rep. (2017) 44:435–40. doi: 10.1007/s11033-017-4121-4 28849415

[22] Ma AS Grigg JR Ho G Prokudin I Farnsworth E Holman K. Sporadic and Familial Congenital Cataracts: Mutational Spectrum and New Diagnoses Using Next-Generation Sequencing. Hum Mutat. (2016) 37:371–84. doi: 10.1002/humu.22948 26694549 PMC4787201

[23] Sun W Xiao X Li S Guo X Zhang Q. Exome sequencing of 18 Chinese families with congenital cataracts: a new sight of the NHS gene. PLoS One. (2014) 9:e100455. doi: 10.1371/journal.pone.0100455 24968223 PMC4072665

[24] Vanita V Singh D Robinson PN Sperling K Singh JR. A novel mutation in the DNA-binding domain of MAF at 16q23.1 associated with autosomal dominant “cerulean cataract” in an Indian family. Am J Med Genet A. (2006) 140:558–66. doi: 10.1002/ajmg.a.31126 16470690

[25] Patel A Hayward JD Tailor V Nyanhete R Ahlfors H Gabriel C. The Oculome Panel Test: Next-Generation Sequencing to Diagnose a Diverse Range of Genetic Developmental Eye Disorders. Ophthalmology. (2019) 126:888–907. doi: 10.1016/j.ophtha.2018.12.050 30653986

[26] Hansen L Eiberg H Rosenberg T. Novel MAF mutation in a family with congenital cataract-microcornea syndrome. Mol Vis. (2007) 13:2019–22 17982426

[27] Narumi Y Nishina S Tokimitsu M Aoki Y Kosaki R Wakui K. Identification of a novel missense mutation of MAF in a Japanese family with congenital cataract by whole exome sequencing: a clinical report and review of literature. Am J Med Genet A. (2014) 164:1272–6. doi: 10.1002/ajmg.a.36433 24664492

[28] Ma A Grigg JR Flaherty M Smith J Minoche AE Cowley MJ. Genome sequencing in congenital cataracts improves diagnostic yield. Hum Mutat. (2021) 42:1173–83. doi: 10.1002/humu.24240, PMID: 34101287

[29] Li J Leng Y Han S Yan L Lu C Luo Y. Clinical and genetic characteristics of Chinese patients with familial or sporadic pediatric cataract. Orphanet J Rare Dis. (2018) 13:94. doi: 10.1186/s13023-018-0828-0 29914532 PMC6006596

[30] Hansen L Mikkelsen A Nürnberg P Nürnberg G Anjum I Eiberg H. Comprehensive mutational screening in a cohort of Danish families with hereditary congenital cataract. Invest Ophthalmol Vis Sci. (2009) 50:3291–303. doi: 10.1167/iovs.08-3149 19182255

[31] Ziats MN Ahmad A Bernat JA Fisher R Glassford M Hannibal MC. Genotype-phenotype analysis of 523 patients by genetics evaluation and clinical exome sequencing. Pediatr Res. (2020) 87:735–9. doi: 10.1038/s41390-019-0611-5 31618753 PMC7082194

[32] Vanita V Guo G Singh D Ott CE Robinson PN. Differential effect of cataract-associated mutations in MAF on transactivation of MAF target genes. Mol Cell Biochem. (2014) 396:137–45. doi: 10.1007/s11010-014-2150-z, PMID: 25064449

[33] Fujino M Tagami A Ojima M Mizuno S Abdellatif AM Kuno A. C-MAF deletion in adult C57BL/6J mice induces cataract formation and abnormal differentiation of lens fiber cells. Exp Anim. (2020) 69:242–9. doi: 10.1538/expanim.19-0137, PMID: 31969519 PMC7220708

[34] Imbratta C Hussein H Andris F Verdeil G. C-MAF, a Swiss army knife for tolerance in lymphocytes. Front Immunol. (2020) 11:206. doi: 10.3389/fimmu.2020.00206, PMID: 32117317 PMC7033575

[35] Li L Yue JF Kong DQ Sun MM Li K Zheng GY. Novel cataract-causing variant c.177dupC in c-MAF regulates the expression of crystallin genes for cell apoptosis via a mitochondria-dependent pathway. Mol Gen Genomics. (2023) 298:495–506. doi: 10.1007/s00438-022-01982-3, PMID: 36719481

[36] Anand D Lachke SA. Systems biology of lens development: a paradigm for disease gene discovery in the eye. Exp Eye Res. (2017) 156:22–33. doi: 10.1016/j.exer.2016.03.010, PMID: 26992779 PMC5026553

[37] Reza HM Urano A Shimada N Yasuda K. Sequential and combinatorial roles of maf family genes define proper lens development. Mol Vis. (2007) 13:18–30. PMID: 17262012 PMC2503189

[38] Richards S Aziz N Bale S Bick D Das S Gastier-Foster J. Standards and guidelines for the interpretation of sequence variants: a joint consensus recommendation of the American College of Medical Genetics and Genomics and the Association for Molecular Pathology. Genet Med. (2015) 17:405–24. doi: 10.1038/gim.2015.30, PMID: 25741868 PMC4544753

[39] Lenhart PD Lambert SR. Current management of infantile cataracts. Surv Ophthalmol. (2022) 67:1476–505. doi: 10.1016/j.survophthal.2022.03.005, PMID: 35307324 PMC10199332

[40] Shiels A Hejtmancik JF. Biology of inherited cataracts and opportunities for treatment. Annu Rev Vis Sci. (2019) 5:123–49. doi: 10.1146/annurev-vision-091517-034346, PMID: 31525139 PMC6791712

